# Mucocutaneous Leishmaniasis: Knowledge, Attitudes, and Practices Among Paraguayan Communities, Patients, and Health Professionals

**DOI:** 10.1155/2013/538629

**Published:** 2013-04-15

**Authors:** Mónica Ruoti, Rolando Oddone, Nathalie Lampert, Elizabeth Orué, Michael A. Miles, Neal Alexander, Andrea M. Rehman, Rebecca Njord, Stephanie Shu, Susannah Brice, Bryony Sinclair, Alison Krentel

**Affiliations:** ^1^Universidad Nacional de Asunción, Instituto de Investigaciones en Ciencias de la Salud, Departamento de Ciencias Sociales, 1120 Asunción, Paraguay; ^2^Universidad Nacional de Asunción, Instituto de Investigaciones en Ciencias de la Salud, Departamento de Producción-Bioquímica, 1120 Asunción, Paraguay; ^3^London School of Hygiene and Tropical Medicine, Faculty of Infectious and Tropical Diseases, Department of Pathogen Molecular Biology, Keppel Street, London WC1E 7HT, UK; ^4^Faculty of Epidemiology and Population Health, Department of Infectious Disease Epidemiology, MRC Tropical Epidemiology Group, London School of Hygiene and Tropical Medicine, Keppel Street, London WC1E 7HT, UK; ^5^Faculty of Tropical and Infectious Diseases, London School of Hygiene and Tropical Medicine, Keppel Street, London WC1E 7HT, UK; ^6^University of Alberta, School of Public Health, Edmonton, AB, Canada T6G 2T4

## Abstract

Cutaneous leishmaniasis (CL) and mucocutaneous leishmaniasis (MCL) due to *Leishmania (V.) braziliensis* are endemic in Paraguay. We performed a series of knowledge, attitudes, and practice (KAP) surveys simultaneously with individuals in endemic communities in San Pedro department (*n* = 463), health professionals (*n* = 25), and patients (*n* = 25). Results showed that communities were exposed to high risk factors for transmission of *L. braziliensis*. In logistic regression analysis, age was the only factor independently associated with having seen a CL/MCL lesion (*P* = 0.002). The pervasive attitude in communities was that CL was not a problem. Treatment seeking was often delayed, partly due to secondary costs, and inappropriate remedies were applied. Several important cost-effective measures are indicated that may improve control of CL. Community awareness could be enhanced through existing community structures. Free supply of specific drugs should continue but ancillary support could be considered. Health professionals require routine and standardised provision of diagnosis and treatment algorithms for CL and MCL. During treatment, all patients could be given simple information to increase awareness in the community.

## 1. Introduction

Leishmaniasis is an important cause of disability in 98 endemic countries and 3 territories with 350 million people living at risk of infection [1, 2]. It is estimated that there are between 0.7 to 1.2 million new cases of cutaneous leishmaniasis (CL) and between 0.2 to 0.4 million cases of visceral leishmaniasis (VL) per year [2]. The burden of CL and VL is estimated globally at a loss of just under 2.4 million DALYs [3]. With increasing deforestation, human migration, urbanization, and HIV/AIDS, leishmaniasis is a growing public health concern in many countries [4]. Leishmaniasis disproportionately affects the poor, particularly those with vulnerable housing and environmental conditions. Loss of income and health care costs exacerbate the economic situation of already disadvantaged households [5]. Leishmaniasis is increasingly seen in domestic and urban environments [6]. Surveys from Latin America (Guatemala [7], Ecuador [8], Colombia [9], Peru [10], and Brazil [11–13]) have looked at knowledge, attitudes, and practices (KAPs) and the use of traditional medicine by those with skin lesions. A pilot KAP study of patients and health professionals conducted in 2003 in Paraguay provided some insight and encouraged further research (S. Brice, unpublished data). 

CL is endemic in 22 Latin American countries. In Paraguay, CL has traditionally been endemic in most of the eastern region, especially in San Pedro, Canindeyú, and Alto Paraná departments [14, 15]. During the period 2007–2009, the average number of registered cases was 381 for the country and 69 for San Pedro, with incidences of 0.64 and 20.4 per year per 100,000 inhabitants, respectively [16]. The proportion of cases classified as MCL was an average of 24.5% over the last 10 years, which exceeded rates seen in other countries in the region: unreported cases may be 4 or 5 times official numbers [17, 18]. The disease agent has been identified as *Leishmania (V.) braziliensis*, initially by multilocus enzyme electrophoresis [19] and, from 2002, either by PCR targeting kDNA regions (E. Nara, IICS-UNA, unpublished data) or by PCR-RFLP of the spliced leader miniexon [18]. Sand flies incriminated as vectors in Paraguay include: *Nyssomyia whitmani* (Antunes and Coutinho, 1939), *Migonemyia migonei* (França, 1920) and *Nyssomyia neivai* (Pinto, 1926) [20, 21]. The silvatic reservoir host of CL/MCL in Paraguay has not yet been proven. However, PCR-based detection has recently implicated rodents of the genera *Oryzomys, Calomys, Oligoryzomys *and *Akodon*, and marsupials of the genus *Didelphis* (R. Oddone, unpublished data).

Here we explore the knowledge, attitudes, and practices of endemic communities as well as patients and health professionals to derive recommendations for the improvement of prevention and care. Understanding the impact of leishmaniasis and human behaviour surrounding the disease is crucial to improving control and treatment.

## 2. Materials and Methods

### 2.1. Study Site

 San Pedro department, located northeast of the capital Asunción, was chosen for the community survey because it has one of the highest levels of CL/MCL endemicity in Paraguay (see Map at Figure 3). San Pedro has an estimated population of 353,000 from a total of 6,341,000 in Paraguay (Dirección General de Estadística, Encuestas y Censos, DGEEC). The economy of San Pedro depends largely on livestock (cattle, horses, and pigs) and agriculture (tobacco, soybeans, and wheat). Recent increased deforestation has led to greater livestock production. Interviews were conducted in 3 of 20 endemic districts: San Estanislao, Guayaibí, and Itacurubí del Rosario, in total covering 39 villages. Patients and health professional respondents were from San Pedro, Asunción, and the Central department. 

### 2.2. Data Collection

Interviewers were social scientists from the Instituto de Investigaciones en Ciencias de la Salud (IICS-UNA). The primary researchers participated in a 2-day workshop with researchers who were conducting parallel studies in Brazil, Venezuela, and Peru. A pilot study of 100 individuals from an endemic community, 10 health professionals, and 10 CL/MCL patients was performed in Paraguay to validate the questionnaires, and adjustments were made accordingly. All questionnaires included both closed and open-ended questions and were appropriate and available in Spanish and Guaraní, the national languages of Paraguay.

For the community KAP, interviews were conducted at the household level with one respondent per household and elicited responses concerning:

- *knowledge* of CL/MCL in terms of local names, transmission, symptoms, prevention, and information source;
- *attitudes* whether the disease was considered to be a problem and if so, what kind of problem and for whom;
- *practices* with regards to prevention, treatment seeking behavior, side effects of treatment, and economics of treatment.

Sample size for the community KAP was determined from a pilot survey in Venezuela in July 2006 (F. Malcolm, unpublished data). Intracluster correlation *ρ* (rho) was calculated at 0.25, and a cluster size of 7 with a design effect of 2.5 was used. A cluster was defined as 7 households. The precision of confidence intervals was set to ±7.5% around an estimated proportion of 50%. A sample size of 448 with an added refusal rate of 3% achieved a final sample size of 460. All villages in the three selected districts were considered, with the sample divided amongst them proportional to the total reference population following the Expanded Programme for Immunization (EPI) methodology [22]. For each cluster, seven households were chosen randomly once the interview team entered the village. The person interviewed in each household was then chosen randomly through a selection of random numbers. If no one aged over 14 years was present in the house, then the interviewer proceeded to the next house. 

The patient KAP included open-ended questions on the patient's experience with CL/MCL, mechanisms of receiving information, treatment-seeking behavior, costs of treatment, barriers to treatment, management of side effects, perception of efficacy, and the disease impact on personal, social, and working life. Purposive sampling strategy was used for patient interviews. For inclusion, patients had to have had active CL and/or MCL within the last 2 years. A caretaker responded to the questionnaire on behalf of patients less than 14 years. Patients were identified through existing clinical records; 25 were selected, 14 from San Pedro and 11 from other endemic areas, who were interviewed at the IICS.

The health professionals KAP included open-ended questions on: knowledge of current protocols for treatment, personal experience with treatment, specific training, opinions of patient experiences (costs, side effects, traditional treatments), what would happen if treatment was no longer offered freely, and main obstacles facing CL/MCL treatment. Purposive sampling strategy was also used for the 25 health professional interviews. Health professionals could be doctors, nurses, midwives, nursing assistants or health promoters, providing they had experience of assessing and treating CL/MCL patients. 

### 2.3. Data Analysis

Data from the community KAP were double-entered into EpiData. Validation of the datasets was performed, and the data were cleaned for inconsistencies and then transferred to STATA 8.0 for further analysis. Descriptive, bivariate, and logistic regression analyses were performed. Adjusted odds ratios were calculated to evaluate the independent associations between sex, age, income, education or location, and specific knowledge, attitudes, and practices, adjusting for the clustered survey design [22]. The adjusted Wald test was used to determine statistical significance. Open-ended questions were entered into Excel and were further categorized according to theme. In some instances, these were back coded and entered into STATA 8.0. For the patient and health professional, KAPs data from the closed questions were also entered into EpiData and then transferred to STATA 8.0 for further analysis. Descriptive statistics were performed. The open-ended questions were analyzed within Excel, and the range of salient themes was identified. Scores were calculated for each individual aware of CL (*n* = 250) for the categories of knowledge, attitudes, and practices as well as a composite KAP score (out of a total of 24 points). These scores were analyzed descriptively. 

Ethical approvals were from the Ethical-Scientific Committee at the IICS-UNA in Paraguay and the ethical committee of the London School of Hygiene and Tropical Medicine. All respondents participated by informed consent form and were assured of confidentiality and anonymity. After each interview, all respondents received printed materials with information about CL/MCL, provided by the Ministry of Public Health.

## 3. Results

### 3.1. Community

A total of 463 people were interviewed: 69% (321/463) from rural areas, 25% (116/463) from peri-urban, and 6% (26/463) from urban areas. Respondents were aged between 14 and 87 years with a mean of 40 years (Table 1). The agricultural sector accounted for 50% (230/463) of employment whilst 26% of respondents (122/463) were housewives. Most households had electricity (96%, 445/463) and water from a piped supply (74%, 342/463). Indicators of higher socioeconomic status were determined to be cement floors (32%, 148/463), tiled floors (19%, 89/463), and ownership of cows (58%, 266/463) horses (24%, 112/463), or pigs (75%, 346/463). Soil floors (29%, 134/463) and using a well as the primary water source (14%, 66/463) were indicators of a lower socioeconomic status. 

#### 3.1.1. Respondent Risk for CL/MCL

Rodents, marsupials, and dogs, possible reservoirs for CL/MCL, were reported to live within 20 paces of 81% (376/463: dogs) and 40% (184/463: rats) of households. Just over 80% (374/463) owned a dog, and for 7% (25/374) had their dog sleep inside the house. Five percent (22/462) of respondents lived within 20 paces of rubbish piles. Whilst most of the population slept in beds indoors (99%, 462/463), bed net use every night was low (7%, 31/463), with 86% (397/463) never having used a bed net. 

#### 3.1.2. Knowledge Concerning CL/MCL

When shown a photograph, 29% (135/463) of respondents had previously seen a CL/MCL lesion, while half (50%, 232/463) had heard of CL/MCL (*Llaga* in Spanish and *Kuruvai* in Guaraní). From both questions, 213 respondents had neither seen a CL/MCL lesion nor heard about CL/MCL and were, therefore, excluded from dependent questions concerning CL/MCL; questions relating specifically to CL/MCL were pursued with the remaining 250. For these respondents knowledge of CL/MCL was moderate: 70% (176/250) understood that everyone can get CL/MCL but 38% (94/250) believed direct transmission of CL/MCL from person to person was possible. Most respondents believed that dogs 36% (91/250), sand flies 29% (72/250), mosquitoes 8% (20/250), or other insect bites 35% (87/250) were responsible for transmission (more than one answer possible). The mean KAP score for knowledge was 5.6 (from a total score of 15), and the range was 1–12 (see Table 2). 

**Figure 1:**
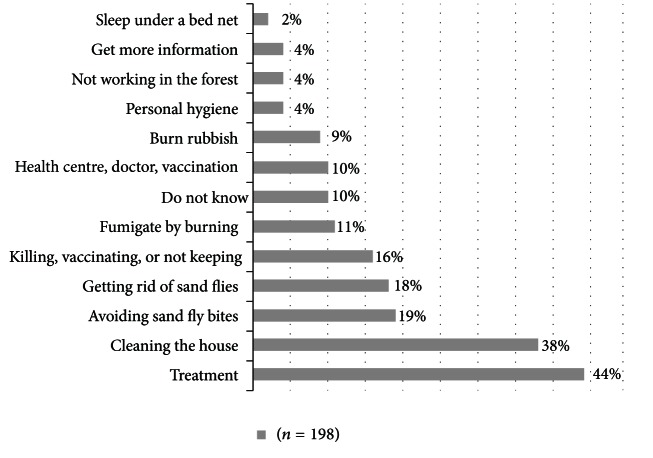
Methods of prevention of leishmaniasis stated by endemic community respondents.

Just over half of respondents (55%, 137/250) named skin lesions as a symptom of CL/MCL and 14% (36/250) cited lesions of the nose and mouth. Thirty percent (76/250) did not know any signs or symptoms of the disease. Only a quarter of the sample (62/250) knew that one could have CL/MCL and be asymptomatic. Most respondents heard about CL/MCL from their family, friend, or neighbour (57%, 143/250). Other sources of awareness and information included: television (38%, 96/250), radio (30%, 76/250), community health worker (12%, 29/250), school (6%, 15/250), and brochure or poster (1%, 3/250) (more than one response considered). The majority of respondents believed that CL/MCL could be prevented (79%, 198/250) (Figure 1). 

#### 3.1.3. Attitudes

KAP scores for attitude were moderate, and the mean for 250 respondents was 3.1 (a perfect score was 7) (see Table 2). Respondents were asked who they thought was most likely to get CL/MCL. Ten percent (25/250) agreed that CL/MCL was a problem in their area, while 74% (184/250) said that it was not, and a further 16% (41/250) did not know. A hypothetical household postulated in the questionnaire (a mother, father, girl child, boy child, and baby) was used to discern respondents' perceptions as to who had priority for CL/MCL treatment. Children (regardless of sex) (40%, 100/250) were perceived to have the highest priority, followed by equality of all (21%, 53/250), then babies (17%, 43/250) and adults (14%, 37/250). 

#### 3.1.4. Experience with CL/MCL

**Figure 2:**
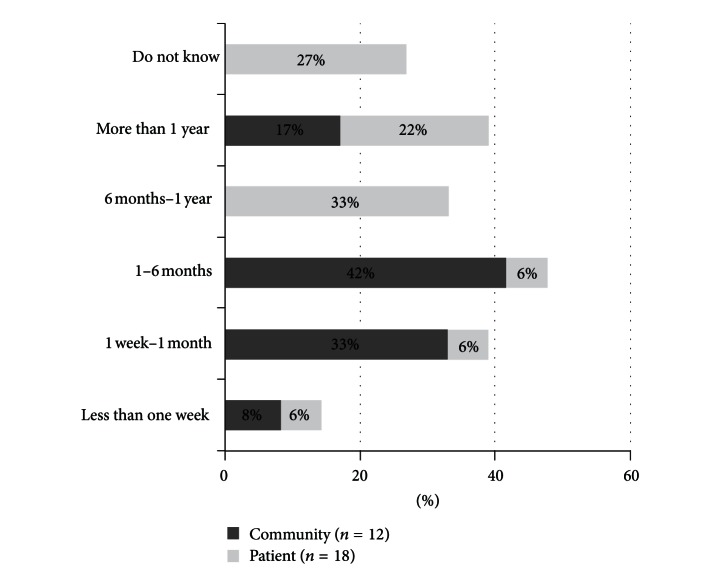
Delay between onset of symptoms and treatment seeking by community and patient respondents.

**Figure 3:**
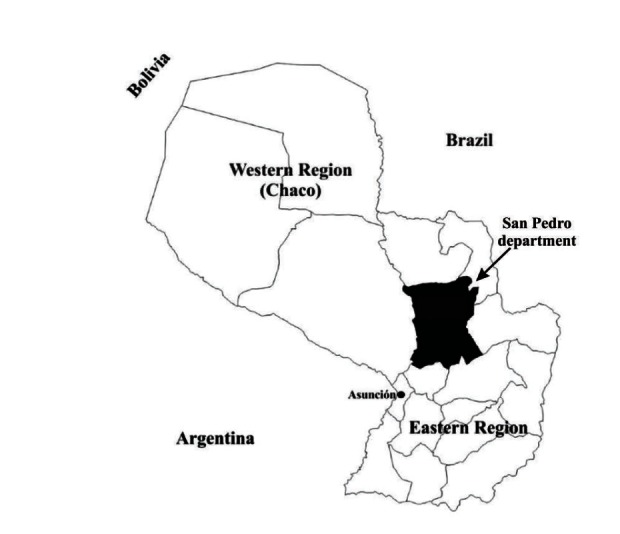
Map.

Fifteen people out of 250 (6%) reported that member(s) of their families had been infected with CL/MCL within the previous two years. Fourteen infected family members sought help for CL/MCL, primarily from the hospital (8/14) followed by the pharmacy (4/14), local health centre (2/14), family, friend, or neighbour (2/14), and traditional healers (2/14) (more than one answer possible). Twelve community respondents reported being personally infected with CL/MCL, with time of onset of symptoms and treatment seeking between 1–6 months (42%; 5/12) (Figure 2). Eleven reported receiving treatment in the form of many injections over time. Only 3 people received information about leishmaniasis at the time of treatment. 

#### 3.1.5. Logistic Regression Analysis

Age was the only factor independently associated with having seen a CL/MCL lesion (*P* = 0.002) adjusting for sex, income, location of house, and education. The odds of having seen a CL/MCL lesion increased with age, relative to those aged 14–24, such that those aged 35–44 years had OR 3.4 (95% CI: 1.6–7.1), those aged 45–59 years had OR 4.2 (95% CI: 1.9–8.9), and those aged 60 years and above had OR 4.1 (95% CI: 1.7–9.7). Factors independently associated with having heard of CL/MCL were age, education, location of house, and income (Table 3). 

Respondents were asked which interventions they would accept if the health authorities were to introduce them. They were most likely to accept outdoor residual spraying (81%, 375/463), environmental clean-up (73%, 338/463), indoor residual spraying (52%, 241/463), treated dog collars (31%, 142/463), and insecticide treated bed nets (28%, 130/463) (Table 4). 

### 3.2. Patients

Twenty-five patients or their carers were interviewed (Table 1). Most (60%, 15/25) were farmers while 12% (3/25) were housewives, 8% (2) traders, 4% (1) labourers, 4% (1) in private business, and 4% (1) military or police. Many patients (44%, 11/25) travelled to their health centre by walking. Other modes of transportation included public bus (28%, 7/25), motorbike (12%, 3/25), private cars or taxi (8%, 2/25). Using the stated form of transportation 64% (16/25) patients lived within 1 hour of the health centre while 32% (8/25) lived 1–3 hours away. Nearly three quarters (18/25) of the patients received water through a piped supply, while 16% (4/25) used a hand pump and well. Thirty-six percent (9/25) of the patients had floors made of soil, whilst 28% (7/25) had cement flooring. 

#### 3.2.1. Knowledge

Nearly all patients (96%, 24/25) reported having seen a CL/MCL lesion when shown a photograph while 76% (9/25) had heard the term for cutaneous leishmaniasis (*Llaga*, *Kuruvai*). When asked specific questions about CL/MCL, only 6/25 patients identified sand flies as responsible for CL/MCL transmission. Nearly three quarters (18/25) knew that CL/MCL could be prevented. Twenty-eight percent (7/25) knew that one could be asymptomatic with CL/MCL. 

#### 3.2.2. Attitudes

Just over half of patients (56%, 14/25) reported that CL/MCL was a problem in their area. When asked why it was a problem, the range of responses included associations with CL/MCL cases they had seen, new settlements, indigenous settlements, the interior of the country, and some named specific localities. When asked what would happen without treatment 7/25 believed they would have become increasingly sick, 1/25 that they would have been scarred, and 13/25 that they would have died. 

#### 3.2.3. Experiences with CL/MCL

Time between onset of symptoms and treatment-seeking ranged from less than one week (2/24) to more than one year (4/24), with most patients waiting between 1–6 months (11/24) (Figure 2). Two patients did not know when they sought treatment. When asked why they waited to seek treatment, patients predominantly reported lack of information about CL/MCL or not having enough funds. Six patients were worried that the lesion would not heal, and three were ashamed of their wound. Affects on daily life were, loss of work, emotional distress, physical symptoms and increased anxiety. Some patients sought treatment from health personnel (8/25) but others (6/25) treated their lesions at home with salt water and herbal remedies, gargling, or by putting gunpowder on the ulcer. Proximity to health centre, information received, treatment/care given, success of treatment, free treatment, and comfortable environment were factors determining patients' treatment-seeking behavior. During treatment patients reported weakness and lethargy (9), fever (2), headache (2), dizziness (2), fear of pain of injection (1), and stomach ache (1) but a third (8) felt no specific side effects. 

The cost of treatment borne by the patient (drugs, time lost, transport, health centre accommodation) varied from no costs (5) to 20,000–700,000 Paraguayan Guaraní (PYG) (13) to over 10,000,000 PYG ($2,358 USD) (3)—nearly half of the average per capita income ($5,200 USD). Mean total cost of treatment was 1,970,476 PYG ($465 USD), and median total cost was 300,000 PYG ($71) (1 USD = 4,240 PYG).

### 3.3. Health Professionals

Most of the 25 health professionals were nurses or auxiliary nurses (13/25); 11/25 were doctors; 8 were from Asunción and the Central department; 10/25 had access to the Internet, and 15/25 had access to medical journals; 24/25 would have liked more information about leishmaniasis (Table 1). 

#### 3.3.1. Knowledge

Nearly all of the health professionals when shown a photo of a lesion had seen CL/MCL before (23/25). Most described it as an illness transmitted by sand flies (21/25), dogs (3/25), and mosquitoes (3/25). There was a fairly consistent understanding that lesions appeared with impact on the skin, in particular the nose and mouth. Some professionals (2/25) reported that leishmaniasis was transmitted from person to person. Most respondents (17) reported that without proper treatment the patient's condition would worsen and five stated that it could be fatal. 

#### 3.3.2. Attitudes

When asked if CL/MCL is a problem in the area, 10 respondents answered yes, 12 said no, and 3 did not know; those agreeing that CL/MCL was a problem cited patient-focused reasons such as economic barriers (diagnosis and treatment are expensive) and that people were not treated. Other reasons given included deforestation and the need for fumigation to eliminate leishmaniasis. 

#### 3.3.3. Experience with CL/MCL

All 25 respondents were asked to describe the protocol for diagnosis and treatment of CL/MCL. Most began with an examination of the ulcer itself; 14 who had the capacity would use the Montenegro skin test; 4 would use biopsy smears for diagnosis. Two mentioned, the immunofluorescence test and two the PCR test, although not always available. Four respondents said that they did not know how to do the diagnosis. Over half (13) of the health professionals have had previous experience treating leishmaniasis. These 13 form the denominator for the remaining questions on treatment. These health staff treated primarily CL/MCL (10), with 1 treating VL only and 2 treating both CL and VL. 

The 13 experienced respondents offered drugs for free and concluded that otherwise people would not follow the treatment because they could not afford medication. Four respondents referred patients to the hospital or health centre for treatment and drugs mentioned to treat leishmaniasis included Glucantime, Pentostam, antimonials in general, and Amphotericin B for MCL. When asked what costs patients incur, most (10) cited transportation costs to and from the hospital and several others mentioned maintenance and accommodation, consultation, other medications, or loss of income. 

## 4. Discussion

This research provides an extensive detailed analysis of perceptions and behavior surrounding prevention, treatment, and knowledge of CL and MCL in San Pedro department, Paraguay. Although the data collection was concentrated primarily in one department, results and analyses can be extrapolated to other parts of the country with similar socioeconomic characteristics. This is the first study in Paraguay to assess the KAP of endemic communities, patients, and medical professionals simultaneously, with a substantial community sample. Due to the security situation, interviewers could not conduct their research at night: a sampling limitation for the community survey was, therefore, biased towards female respondents (296 : 167). 

Leishmaniasis is spread by some 30 different sand fly species and affects a wide variety of hosts including humans, dogs, horses, and foxes [3, 23, 24]. Rodents are considered to be one of the possible reservoirs for leishmaniasis in Paraguay and over a quarter of patients and nearly half of community members lived within 20 paces of rats. Chicken and dog ownership was high in both surveys (>70% in both surveys); chickens sustain and propagate sand fly populations. Dogs have not been shown to be a domestic reservoir of *Leishmania (Viannia) braziliensis* in Paraguay but they have been implicated elsewhere [25, 26]. Other risk associated environmental features include proximity to forest and rubbish piles [27] and soil in animal shelters [28]; around a third of patients and of community respondents had soil floors, a possible sand fly breeding zone [27]. In Paraguay, sand fly density has been correlated to the density of vegetation coverage, and the diversity of species is associated with proximity to primary forest. Male to female ratio in the patient survey was 18 : 7, consistent with men having greater risk due to occupation and increased exposure to the vector [29, 30]. Environmental changes around the house, such as burying rubbish, putting the dog outside to sleep, and keeping chickens and other animals in open structured shelters further from the house, are theoretically within the power of the homeowners if they are informed and understand the importance of taking these precautionary measures. 

Despite the risk factors, regular use of bed nets was low among both patient, and community respondents (16% and 7%, resp.). Recent studies from Iran and Venezuela indicate that consistent use of insecticide treated bed nets and curtains provide some personal protection against sand fly bites and transmission of zoonotic CL [6, 31]. Low use of bed nets may be due to lack of knowledge on their relevance to prevention of leishmaniasis as well as the concept of prevention is not always prioritized by people in Paraguay (M. Ruoti, unpublished data). Low bed net use may also be attributed to local attitudes as the data revealed that those in peri-urban areas with increased income were less likely to use bed nets as an intervention (see Table 4). Community and patient education campaigns may consider including more purposeful prevention efforts, including enhancing access to insecticide treated bed nets and curtains in areas endemic for both malaria and leishmaniasis. Careful attention should be paid to the cultural and social context of the intended audience, for example, whether rural or peri-urban. 

In all three surveys, respondents consistently mentioned costs associated with treatment of leishmaniasis. Although antimonial drugs are offered at no cost in Paraguay, not all costs associated with treatment are included in current government policy. Among the additional costs patients reported were, consultations, related medical supplies and antibiotic therapy for secondary infections. Collateral costs such as loss of income, travel, and maintenance costs during treatment also impose an economic burden and may affect followup of cases. Patients may weigh the financial risk of seeking treatment with the impact on their vulnerable household economy. This may well affect the number of cases recorded, increase time between onset of symptoms and treatment, and increase the number of mucosal cases. Our findings suggest that a holistic policy approach to treatment needs to be explored, which includes cost recovery mechanisms to ease the financial burden that treatment can bring to disadvantaged populations. For example, enlisting a specific agency or company to transport patients by motorbike to their closest health post could ease the transportation burden for some patients, proving to be a highly cost effective measure. A holistic policy approach to treatment would save the enormous costs associated with complex treatment and clinical management of late cases. 

One clear finding of this research is the need for increased and consistent awareness building activities in endemic communities. At the community level, just over 50% were aware of CL/MCL and specific knowledge of the disease and its transmission, and prevention was moderate. Some misinformation was apparent, the concept that leishmaniasis can be passed from person to person, an echo of a pilot patient and health professional KAP in Paraguay (S. Brice, unpublished data). This misconception may augment stigma and hinder CL and MCL sufferers from accessing assistance and care.

Furthermore, across the three surveys, less than half of respondents (in the community survey only 10%) agreed that leishmaniasis was a problem in their area. Awareness activities need to describe frequency of disease, the cost and duration of treatment, and severity of metastatic disease (MCL). Those living in endemic communities could also be better informed on transmission by sand flies, prevention methods, personal risk, how to identify a skin, lesion and where and when to seek treatment. Older people may be useful to incorporate in educational activities as they have a higher awareness level than their younger peers and may command a degree of respect in community outreach. Education could acknowledge differential risks for men and women, their roles and responsibilities for health. For example, messages to women might include treatment seeking information and environmental clean up, which could be undertaken at the household level, whereas men could learn about occupational exposure, prevention of sand fly bites and recognition of a skin lesion. Based on our data, radio is the preferred means for mass media campaigns, because TV ownership is moderate and newspaper readership is low. Other avenues for education and awareness activities include churches and elementary schools. Most community members and patients received their information from family members, friends, or neighbours, confirming earlier findings (S. Brice, unpublished data). Community mobilization programs need to incorporate and activate such existing social networks to maximize information dissemination. 

Most patients did not receive information at time of treatment. Health professionals are recommended to take advantage of consultation to inform and assist patients to understand their condition, treatment, and how to prevent it in the future. Simple posters are recommended to assist medical personnel in the clinics, and simple brochures with pictures for patients to take home to families and neighbours. It is also suggested that the National Control Programme follow-up on the extent of distribution of printed materials for patients, health professionals, and communities. Commonly materials are produced but may not actually reach their target audiences due to distribution constraints, turnover of staff, lack of funds, and low priority of prevention (A. Krentel, unpublished data). Another means of communication to consider is the use of mobile phone technology to remind patients of follow-up appointments. Incomplete treatment is a serious hazard for the development of drug resistance. Increased patient education and financial facilities to aid disadvantaged patients will reduce the rate of patients not completing treatment.

Health professionals displayed sufficient general information about leishmaniasis. However, it appears from their responses that there is a need for a streamlined approach to diagnosis of CL/MCL in Paraguay, paying particular attention to the level of health care facility, for example, what may be feasible at the primary health care centre may not be at a smaller auxiliary health post. In this respect, training of nurses and health promoters may also maximize the diagnosis and treatment of CL/MCL; algorithms for diagnosis and treatment could be made widely available. Methods of communication (internet, mobile phone technology) with health professionals could also be enhanced to keep them updated and demonstrate the importance of leishmaniasis as a growing concern in their communities. 

## 5. Conclusion

As both CL and MCL are increasing in many countries globally, including Paraguay, the policy implications and recommendations discussed here are relevant in a wider context. Specifically, attention could be given to implementing cost effective measures that are likely to improve the timely treatment of CL and MCL patients, thus avoiding higher costs associated with complex treatment and clinical management of late cases. Government and private agencies need to be aware of the cost implications of treatment, even when antimonials are offered at no cost and could make cost recovery programs a priority to improve treatment seeking behavior and compliance with treatment regimens. Communities affected by CL and MCL could be prioritized for appropriate awareness campaigns, including lesion recognition, prevention, and information on treatment. Awareness campaigns could utilize the existing structures within the community, for example, schools, churches, and civil organizations and include older people and past patients in educational activities. By implementing the cost effective measures discussed in this paper, prevention of new cases, better case recognition, improved treatment-seeking behavior, and sustained compliance with treatment can be an attainable goal. 

## Figures and Tables

**Table 1:** Characteristics of samples from endemic community, patients, and medical professionals.

	Endemic community	Patients	Medical professionals
Total number of respondents	463	25	25
Mean age of respondents in year (standard deviation)	40 (sd: 16)	49 (sd: 23)	37 (sd: 11)

	% (number)	% (number)	% (number)

Gender			
Male	36% (167)	72% (18)	40% (10)
Female	64% (296)	28% (7)	60% (15)
Education			
None	3% (12)	12% (3)	4% (1)
Primary	65% (302)	72% (18)	32% (8)
Secondary	27% (125)	12% (3)	32% (8)
University	5% (24)	4% (1)	32% (8)
Monthly household income*			
<1 minimum salary ($288 USD)	34% (158)	44% (11)	—
1+ minimum salaries	11% (50)	16% (4)	—
No fixed income	54% (250)	40% (10)	—
No response	1% (5)	(0)	—
Time in current location			
<1 year	4% (20)	4% (1)	20% (5)
1–3 years	8% (35)	8% (2)	28% (7)
3+ years	88% (408)	88% (22)	52% (13)

*1,219,795 PYG at the time of the survey (1 USD = 4240 PYG).

**Table 2:** KAP scores for individuals aware of CL/MCL.

	(*n* = 250)	
KAP scores (total score possible)	*n*	%
Knowledge (15)		
	76	30.4
5–8	155	62
9–12	19	7.6
Attitudes (7)		
1-2	112	44.8
3-4	87	34.8
5–7	51	20.4
Preventive practices (4)		
1-2	33	13.2
3-4	217	86.8
Composite KAP score (24)		
5–9	52	20.8
10–14	163	65.2
15–19	35	14

**Table 3:** Crude and adjusted odds ratios (ORs) and 95% CI and adjusted Wald *P* values of factors affecting having heard of CL/MCL among endemic community respondents.

Risk factor	Crude OR (95% CI)	Adjusted OR* (95% CI)
Age	(*P* = 0.05)	(*P* = 0.008)
14–24 years	1	1
25–34 years	1.54 (0.75–3.17)	1.75 (0.76–3.99)
35–44 years	1.46 (0.75–2.86)	1.95 (0.96–3.98)
45–59 years	2.13 (1.15–3.94)	**2.71 (1.46**–**5.03)**
60+	2.13 (1.24–3.65)	**2.79 (1.51**–**5.17)**

Education	(*P* = 0.69)	(*P* = 0.04)
No education and primary school	1	1
Secondary school	1.09 (0.64–1.87)	1.67 (0.98–2.83)
University	1.45 (0.59–3.61)	2.23 (0.98–5.11)

Sex	(*P* = 0.03)	(*P* = 0.07)
Male	1	1
Female	0.68 (0.48–0.96)	0.72 (0.49–1.04)

Location	(*P* = 0.006)	(*P* = 0.007)
Urban	1	1
Peri-urban	1.70 (0.91–3.18)	1.75 (0.98–3.11)
Rural	2.66 (1.51–4.68)	**2.86 (1.54**–**5.31)**

Income	(*P* = 0.02)	(*P* = 0.02)
<1 minimum salary ($288 USD)	1	1
1+ minimum salary	2.15 (1.21–3.79)	**2.22 (1.21**–**4.07)**
No fixed income	1.73 (1.05–2.86)	**1.78 (1.05**–**3.01)**

*Each adjusted for the other exposure variables: age, income, sex, house location, education.

**Table 4:** Crude and adjusted odds ratios (ORs) and 95% CI and adjusted Wald *P* values of factors affecting acceptance of public health interventions.

Risk factor	Crude OR (95% CI)	Adjusted OR* (95% CI)
Insecticide treated bed nets		

Location	(*P* = 0.09)	(*P* = 0.02)
Urban	1	1
Peri-urban	0.39 (0.16–0.97)	0.29 (0.12–0.68)
Rural	0.87 (0.44–1.72)	0.72 (0.38–1.39)
Income	(*P* = 0.11)	(*P* = 0.05)
<1 minimum salary	1	1
1+ minimum salary	0.45 (0.21–0.95)	0.39 (0.18–0.82)
No fixed income	0.75 (0.46–1.21)	0.79 (0.48–1.29)

Insecticide treated dog collars		

Income	(*P* = 0.02)	(*P* = 0.009)
<1 minimum salary	1	1
1+ minimum salary	0.39 (0.21–0.77)	0.34 (0.17–0.67)
No fixed income	0.77 (0.50–1.17)	0.84 (0.52–1.35)
Education	(*P* = 0.05)	(*P* = 0.014)
No education and primary school	1	1
Secondary school	1.49 (1.07–2.08)	**1.65 (1.15**–**2.37)**
University	2.24 (0.97–5.18)	**2.56 (1.09**–**6.01)**

Environmental clean-up		

Age	(*P* = 0.05)	(*P* = 0.10)
14–24 years	1	1
25–34 years	0.86 (0.41–1.79)	0.82 (0.43–1.58)
35–44 years	0.79 (0.42–1.47)	0.83 (0.39–1.74)
45–59 years	0.58 (0.31–1.05)	0.54 (0.26–1.09)
60+	0.36 (0.17–0.75)	0.34 (0.13–0.84)
Location	(*P* = 0.06)	(*P* = 0.07)
Urban	1	1
Peri-urban	1.93 (0.72–5.15)	1.82 (0.72–4.62)
Rural	2.14 (1.14–3.98)	**2.11 (1.12**–**3.98)**

Indoor residual spraying		

Income	(*P* = 0.006)	(*P* = 0.008)
<1 minimum salary	1	1
1+ minimum salary	0.94 (0.53–1.67)	0.93 (0.54–1.59)
No fixed income	0.50 (0.34–0.75)	0.51 (0.34–0.77)

*Each adjusted for the other exposure variables: age, income, sex, house location, education.
